# Alcohol-assisted versus Mechanical Epithelium Removal in Photorefractive Keratectomy

**Published:** 2010-10

**Authors:** Mohammad Ghoreishi, Hossein Attarzadeh, Mehdi Tavakoli, Heidar Ali Moini, Alireza Zandi, Amin Masjedi, Akram Rismanchian

**Affiliations:** 1 Ophthalmology Department, Isfahan University of Medical Sciences, Isfahan, Iran; 2 Ophthalmic Research Center, Shahid Beheshti University of Medical Sciences, Tehran, Iran

**Keywords:** Photorefractive Keratectomy, Lasers, Excimer, Epithelial Debridement, Ethanol

## Abstract

**Purpose:**

To compare the outcomes and complications of alcohol-assisted versus mechanical corneal epithelial debridement for photorefractive keratectomy (PRK).

**Methods:**

This randomized controlled trial included 1,250 eyes of 625 patients undergoing PRK for correction of myopia and myopic astigmatism. Each patient was randomly assigned to alcohol-assisted or mechanical epithelial removal.

**Results:**

A total of 658 eyes underwent alcohol-assisted epithelial removal while the epithelium was removed mechanically in 592 eyes. Mean spherical equivalent was −4.37±2.3 D in the alcohol group and −3.8±1.3 D in the mechanical group (P = 0.78). There was no significant difference in postoperative pain between the study groups (P = 0.22). Uncorrected visual acuity ≥ 20/20 and ≥ 20/40 was achieved in 90.9% versus 93.4% (P = 0.08), and 98.9% versus 99.5% (P = 0.36) of eyes in the alcohol and mechanical groups, respectively. Final refractive error within 1D of emmetropia was achieved in 90% versus 92.2% of eyes in the alcohol and mechanical groups, respectively (P = 0.23). Alcohol-assisted debridement required less time than mechanical debridement (96±18 vs. 118±26 seconds, P=0.035). There was no significant difference between the two groups in terms of early and late postoperative complications.

**Conclusion:**

Alcohol-assisted and mechanical epithelium removal are comparable in terms of efficacy and side effects. The method of epithelial debridement in PRK may be left to the surgeon’s choice.

## INTRODUCTION

Photorefractive keratectomy (PRK) is a surface ablation procedure with a long history of application for correction of myopia and myopic astigmatism.^1^ Although laser in situ keratomileusis (LASIK) surpassed PRK in the past decades, a new trend favoring PRK and other surface ablation techniques has recently emerged due to certain complications associated with LASIK.^2^–^4^

Before stromal ablation in PRK, the corneal epithelium must be removed. Several techniques of epithelial debridement have been tried, including mechanical debridement, alcohol-assisted debridement, transepithelial laser ablation, and a rotating brush.^5^–^7^ With any of these methods, the epithelium should be removed consistently to prevent hydration changes in the stroma, because the amount of excimer laser ablation may be increased by excessive corneal stromal dehydration, resulting in overcorrection. The most commonly used techniques are simple mechanical and alcohol-assisted removal.

The initial method for epithelial removal was mechanical debridement. Although this method is straightforward and effective, there are drawbacks. Manual epithelial debridement using sharp scalpel blades has been reported to create scratches and nicks in Bowman’s membrane and to leave varying amounts of epithelium.^8^ Residual epithelium and basement membrane may influence the depth of ablation by the excimer laser. Furthermore, especially for inexperienced surgeons, the time required for mechanical debridement can be lengthy. This may cause patient anxiety and reduce stromal hydration due to evaporation.^9^

Alcohol-assisted removal is easier and faster, and probably more comfortable for the patient and surgeon, but may entail toxic side effects.^10^,^11^ Common problems of epithelial debridement include moderate to severe pain, a relatively long period of visual recovery, and corneal haze. The application of alcohol makes epithelial removal simple, fast and complete,^12^,^13^ but entails certain problems. High concentrations of ethanol have been found to cause inflammation and damage to underlying stromal keratocytes.^10^,^14^ Ethanol may also affect stromal hydration.^10^

In this study we compare the safety, efficacy, and predictability of PRK with alcohol-assisted removal versus simple mechanical removal using a blunt hockey blade. We also compare the short term and long term complications with these two techniques.

## METHODS

This randomized controlled trial was performed on 1,250 eyes of 625 patients. All subjects were at least 18 years old and of good ocular and physical health. None had signs of corneal or anterior segment pathology, keratoconus, eyelid disease, uncontrolled glaucoma, untreated retinal abnormalities, progressive or unstable myopia, or previous intraocular or corneal surgery. The risks and benefits of non-surgical and surgical alternatives to PRK were discussed before enrollment. Informed consent was obtained from all patients prior to entering the study. The procedures were randomly assigned to the patients using a random number table. In each group, epithelial removal was performed after application of alcohol or simply mechanically using a hockey blade.

All operations were performed by a single surgeon (MG) and the same excimer laser machine (Technolas 217z100, Bausch & Lomb, Rochester, NY, USA). After instillation of tetracaine eye drops twice within a 5 minute interval, the eyes were exposed using a wire lid speculum. In the mechanical group the epithelium was removed manually in a centripetal fashion using a blunt hockey blade. In the alcohol group, the cornea was exposed to 20% ethylic alcohol for 15 seconds with the aid of a well. The diameter of epithelial removal was 8 mm. The time required or epithelial removal was recorded for each procedure. After epithelial removal, excimer laser ablation was performed. Following laser ablation, 0.02% mitomycin C was applied on the ablated stroma in eyes with myopia more than −4.00 D or astigmatism exceeding 2.00 D. The duration of mitomycin C application was 20 seconds. Eyes were irrigated with chilled balanced salt solution and a bandage contact lens (Bausch & Lomb, Rochester, NY, USA) was placed on the cornea. Ciprofloxacin eye drops were instilled, and the patients were discharged with a prescription of ciprofloxacin 1% eye drops every 4 hours, betamethasone 0.1% every 4 hours, diclofenac eye drops every 6 hours, and artificial tears as needed.

Patients were examined every day in the first postoperative week to evaluate epithelial healing, and at 1, 3, and 6 months. Betamethasone and ciprofloxacin were used for 10 days and then substituted with fluorometholone for 3 weeks which was tapered depending on clinical findings. In special situations such as corneal haze, regression, or increased intraocular pressure the steroid dose was adjusted accordingly. Diclofenac was used only for 2 to 3 days.

Early (less than one month) postoperative findings such as pain, burning, tearing, foreign body sensation, and time for complete epithelialization were recorded. If the healing was not complete after 7 days, it was considered as delayed. In order to evaluate postoperative pain, patients were asked to report pain intensity in each eye on a scale of 0 to 10 using the 11-point numeric scale of pain. This measurement was performed on day 4 postoperatively. The questioning physician first explained to the patients that 0 represented no pain at all and 10 would be the worst imaginable pain. The pain score was recorded separately for the right and left eyes. Re-epithelialization was diagrammed and estimated in percentages for comparison.

Late postoperative findings, such as uncorrected visual acuity (UCVA), best corrected visual acuity (BCVA), and refractive error were measured 6 months after the procedure. Late onset symptoms, such as halos, blurred vision, glare, monocular diplopia, and dry eye symptoms were evaluated. Corneal haze and recurrent corneal erosions were also recorded. Haze levels were determined using a slitlamp according to the method described by Hanna modified by Helena et al.^15^

Statistical analysis was performed using SPSS 11.0 software (SPSS Inc., Chicago, IL, USA). Changes in manifest refraction 6 months after surgery were compared using unpaired t-tests. The percentage of eyes with UCVA of 20/20 or better and 20/40 or better and the percentage within ±0.5 and +1.00 D of emmetropia were compared between the two groups using the chi-square test. Chi-square test was also used to compare frequency data between the two groups. For other comparisons the student t-test or Fisher’s exact test was applied. P-values less than 0.05 were considered as statistically significant.

## RESULTS

Overall, 1,250 eyes of 625 patients including 431 (69%) female and 194 (31%) male subjects with mean age of 28±4 (range, 18 to 55) years were included. Overall, 658 eyes were assigned to the alcohol group and 592 eyes were allocated to the mechanical group. All patients were followed for at least 6 months.

Preoperative refractive error ranged from −0.75 to −9.4 D overall, with a mean value of −4.25±2.27 D. Mean preoperative spherical equivalent refractive error was −4.37±2.3 D in the alcohol group versus −3.8±1.3 D in the mechanical group (P=0.78). Preoperatively, 90.3% versus 90.9% (P=0.96) of eyes in the alcohol and mechanical groups had spherical equivalent refractive error less than −6.00 D (Table 1).

Mean time for alcohol-assisted epithelial removal was 96±18 seconds and that for simple mechanical removal was 118±26 seconds (P=0.035).

Early postoperative complications (presenting during the first month following the operation) were comparable between the study groups and are detailed in table 2. The mean period for re-epithelialization was 4.2±1.6 days in the alcohol group versus 3.8±1.3 days in the mechanical group (P=0.75).

Mean pain score was 4.7±1.2 in the alcohol group versus 5.3±1.5 in the mechanical group (P=0.22). Table 3 further details pain scores in the study groups.

The study groups were comparable in terms of late onset (evaluated 6 months after PRK) complications. The prevalence of late complications is presented in table 4. We observed no case of recurrent corneal erosion. The intensity of corneal stromal haze was mild in the majority of eyes (grade 1 and 2) and was visually significant (grade 3) only in 3 eyes including 2 eyes in the alcohol group and 1 in the mechanical group. Visual acuity and manifest refractive errors in the two study groups at 6 months are summarized in table 5.

## DISCUSSION

In this study we compared the efficacy and safety of alcohol-assisted epithelial debridement with that of simple mechanical removal in PRK.

The procedure was faster in the alcohol group. Such results have also been reported by Carones et al^16^ and Abad et al^17^. The time required for mechanical debridement can be longer than that for laser or alcohol scrape techniques, even in experienced hands.^12^ This may cause stromal dehydration due to the evaporation of fluid and can affect refractive predictability.

In our study we found no significant difference in the prevalence of early onset complications, such as foreign body sensation, burning, glare, and delayed epithelial healing between the study groups. Delayed epithelial healing at day 7 was more common in the mechanical group but the difference was of borderline significance (P=0.07). Abad et al^17^ reported that at 4 days, 95% of alcohol-treated versus 78% of mechanically scraped eyes had healed (P=0.04) and believed that in contrast to alcohol and laser epithelial removal, mechanical removal can produce a rough stromal bed which may hamper epithelial healing. Lee et al^18^ also reported faster epithelial healing in the LASEK group, in which alcohol-assisted epithelial removal had been performed, in comparison to conventional PRK with mechanical epithelial removal. Another study, however reported no significant difference in the rate of re-epithelialization.^12^

In the current series no difference in pain severity was present between the two groups. Similar results have been reported by Lee et al^18^. In contrast, Blake et al^19^ found that patients reported significantly more severe postoperative pain with alcohol-assisted epithelial removal on postoperative day 1, but the difference was not significant by day 3. We did not perform such a comparison in our study.

We detected no difference in the prevalence of late complications, such as late onset corneal haze and dry eye, between the two groups. This is in accordance with Abad et al^12^ and Lee et al^18^, but in contrast to another study reporting less severe corneal haze with alcohol epithelial removal.^16^

There was no significant difference in postoperative visual acuity and refractive error between the two groups which is comparable to several other studies.^13^,^14^,^16^–^18^ In the study by Shah^20^ and colleagues, there was a tendency towards hyperopic shift in patients with alcohol-assisted debridement, however we observed no significant hyperopic shift in either study group and there was no significant difference in this regard between the two methods of epithelial debridement.

In summary, we observed no significant difference between alcohol-assisted and mechanical epithelial debridement for PRK, except that the alcohol method took a few seconds less time. We consider both methods to be comparable in terms of efficacy and complications and believe the choice of one method over the other rests on the surgeon’s decision and experience.

## Figures and Tables

**Table 1:** Baseline features in the alcohol and mechanical groups

	Alcohol group	Mechanical group	P-value
Number of eyes	658	592	–
Preoperative mean spherical equivalent (D)	−4.37±2.3	−3.8±1.3	0.78
Preoperative mean spherical equivalent less than −6 D	90.3%	90.9%	0.96
Preoperative mean spherical equivalent greater than −6 D	9.7%	9.1%	0.71

**Table 2:** Early complications

Complication	Alcohol group	Mechanical group	P-value
Delayed epithelial healing	48 (7.2%)	56 (9.4%)	0.07
Foreign body sensation	65 (9.9%)	69 (11.6%)	0.31
Burning sensation	49 (7.4%)	48 (8.1%)	0.16
Glare	11 (1.7%)	10 (1.7%)	0.95
Blurred vision	40 (6.1%)	41 (6.9%)	0.54

**Table 3:** Severity of pain in the study groups

Pain	Alcohol group	Mechanical group
No pain (pain score of 0)	12 (1.8%)	7 (1.1%)
Mild pain (pain score of 1–3)	262 (39.8%)	205 (34.7%)
Moderate pain (pain score of 4–7)	310 (47.1%)	262 (44.3%)
Severe pain (pain score of 8–10)	74 (11.2%)	118 (19.9%)

**Table 4:** Late complications

Complications	Alcohol group	Mechanical group	P-value
Corneal stromal haze	30 (4.6%)	27 (4.5%)	0.98
Dry eye	73 (11.1%)	65 (11%)	0.95
Blurred vision	38 (5.8%)	33 (5.6%)	0.98
Foreign body sensation	20 (3%)	16 (2.7%)	0.72

**Table 5:** Postoperative visual acuity and refraction in the study groups

	UCVA >20/20	UCVA >20/40	Refraction* ±0.5 D	Refraction* ±1.0 D
Alcohol group	90.9%	98.9%	69.1%	90%
Mechanical group	93.4%	99.5%	69.6%	92.2%

P-value	0.08	0.36	0.07	0.23

UCVA, Uncorrected visual acuity

* Of emmetropia

## References

[1] Biswell R, Riordan-Eva P, Whitcher JP (2004). Cornea. Vaughan & Asbury’s General Ophthalmology.

[2] Hersh PS, Brint SF, Maloney RK, Durrie DS, Gordon M, Michelson MA (1998). Photorefractive keratectomy versus laser in situ keratomileusis for moderate to high myopia. A randomized prospective study. Ophthalmology.

[3] Pop M, Payette Y (2000). Photorefractive keratectomy versus laser in situ keratomileusis: a control-matched study. Ophthalmology.

[4] Kasetsuwan N, Puangsricharern V, Pariyakanok L (2000). Excimer laser photorefractive keratectomy and laser in situ keratomileusis for myopia and astigmatism. J Med Assoc Thai.

[5] Alio JL, Ismael MM, Artola A (1993). Laser epithelium removal before photorefractive keratectomy. Refract Corneal Surg.

[6] Pallikaris IG, Karoutis AD, Lydataki SE, Siganos DS (1994). Rotating brush for fast removal of corneal epithelium. J Refract Corneal Surg.

[7] Johnson DG, Kezirian GM, George SP, Casebeer JC, Ashton J (1998). Removal of corneal epithelium with phototherapeutic technique during multizone, multipass photorefractive keratectomy. J Refract Surg.

[8] Campos M, Hertzog L, Wang XW, Fasano AP, McDonnell PJ (1992). Corneal surface after deepithelialization using a sharp and a dull instrument. Ophthalmic Surg.

[9] Gimbel HV, DeBroff BM, Beldavs RA, van Westenbrugge JA, Ferensowicz M (1995). Comparison of laser and manual removal of corneal epithelium for photorefractive keratectomy. J Refract Surg.

[10] Campos M, Raman S, Lee M, McDonnell PJ (1994). Keratocyte loss after different methods of de-epithelialization. Ophthalmology.

[11] Campos M, Szerenyi K, Lee M, McDonnell JM, Lopez PF, McDonnell PJ (1994). Keratocyte loss after corneal deepithelialization in primates and rabbits. Arch Ophthalmol.

[12] Abad JC, Talamo JH, Vidaurri-Leal J, Cantu-Charles C, Helena MC (1996). Dilute ethanol versus mechanical debridement before photorefractive keratectomy. J Cataract Refract Surg.

[13] Stein HA, Stein RM, Price C, Salim GA (1997). Alcohol removal of the epithelium for excimer laser ablation: outcomes analysis. J Cataract Refract Surg.

[14] Helena MC, Filatov VV, Johnston WT, Vidaurri-Leal J, Wilson SE, Talamo JH (1997). Effects of 50% ethanol and mechanical epithelial debridement on corneal structure before and after excimer photorefractive keratectomy. Cornea.

[15] Hanna KD, Pouliquen YM, Waring GO, Savoldelli M, Fantes F, Thompson KP (1992). Corneal wound healing in monkeys after repeated excimer laser photorefractive keratectomy. Arch Ophthalmol.

[16] Carones F, Fiore T, Brancato R (1999). Mechanical vs. alcohol epithelial removal during photorefractive keratectomy. J Refract Surg.

[17] Abad JC, An B, Power WJ, Foster CS, Azar DT, Talamo JH (1997). A prospective evaluation of alcohol-assisted versus mechanical epithelial removal before photorefractive keratectomy. Ophthalmology.

[18] Lee HK, Lee KS, Kim JK, Kim HC, Seo KR, Kim EK (2005). Epithelial healing and clinical outcomes in excimer laser photorefractive surgery following three epithelial removal techniques: mechanical, alcohol, and excimer laser. Am J Ophthalmol.

[19] Blake CR, Cervantes-Castañeda RA, Macias-Rodríguez Y, Anzoulatous G, Anderson R, Chayet AS (2005). Comparison of postoperative pain in patients following photorefractive keratectomy versus advanced surface ablation. J Cataract Refract Surg.

[20] Shah S, Doyle SJ, Chatterjee A, Williams BE, Ilango B (1998). Comparison of 18% ethanol and mechanical debridement for epithelial removal before photorefractive keratectomy. J Refract Surg.

